# Digital economy development and the urban-rural income gap: Evidence from Chinese cities

**DOI:** 10.1371/journal.pone.0280225

**Published:** 2023-02-28

**Authors:** Xiang Deng, Meng Guo, Yuyan Liu

**Affiliations:** 1 School of Economics, Sichuan University, Chengdu, Sichuan, China; 2 HEOA Group, West China School of Public Health and West China Fourth Hospital, Sichuan University, Chengdu, Sichuan, China; 3 Institute for Health Cities and West China Research Center for Rural Health Development, Sichuan University, Chengdu, Sichuan, China; Northeastern University (Shenyang China), CHINA

## Abstract

The growth of the digital economy has created new forms of inequality of opportunity. This paper studies whether the development of the digital economy expands the income gap between urban and rural areas from theoretical and empirical. The research based on the panel data of 202 cities from 2011 to 2019 in China shows that: (1) Although the digital economy can promote the improvement of both urban and rural absolute income levels, it has a greater positive impact on urban residents’ income levels than on rural residents’, resulting in a widening of the urban-rural income gap. (2) The analysis of the action mechanism reveals that employment in the information service industry and the depth of digital finance use are two crucial mechanisms for the digital economy to widen the income gap between urban and rural areas. (3) The spatial Durbin model(SDM) and the spatial error model(SEM) based on three spatial weight matrices show that the impact of the digital economy on the urban-rural income gap is also characterized by spatial spillover, and the development of the digital economy will also have a negative impact on the urban-rural income gap in neighboring regions as well. (4) The main conclusions still hold after the robustness of quasi-natural experiments based on the strategy of "Broadband China" and the selection of historical data as instrumental variables. This research is helpful to understand the effects, mechanisms and spatial characteristics of digital economy on urban-rural income gap.

## 1. Introduction

According to the Digital Economy Report 2019, the scale of the global digital economy is expected to be 4.5% to 15.5% of global GDP and is still expanding. The digital economy is a brand-new economic structure that uses digital technology as its primary engine to advance the digital transformation and high-quality growth of the world economy through three channels: new technology creates new industries, new industries give rise to new models, and new technology strengthens traditional industries. The impact of digital economy on modern society is profound and extensive. The development of digital economy can not only improve air quality, ease heat island and carbon emissions, enhance climate comfort [1–4], but also bring revolutionary changes to existing business models [5]. With the acceleration of the integration of information technology and real economy, the application of information technology and the Internet constitutes an important factor affecting the efficiency of contemporary economic growth [6]. At the same time, it contributes to the digital divide and deepens the income disparity between urban and rural areas [7]. The international community has been paying attention to the international digital divide since the end of the 20th century, but the situation is still serious after more than ten years. New developments in information technology have widened the international digital divide between countries and made the prospect of social disintegration in many regions increasingly real. The international world is aware of the problem known as the "digital divide," which occurs both inside a nation and between developing nations and developed nations.

Statistics show that the scale of China’s digital economy exceeded US $6.5 trillion in 2021, accounting for nearly 40% of GDP. The roll-out of a series of Internet infrastructure projects and the lowering of the threshold for the use of smart terminals have greatly increased Internet penetration in rural areas, narrowing the gap in access between urban and rural. At the same time, as the credit system, market system, innovation system and other soft infrastructure required for the development of the digital economy are still very imperfect in rural areas, the secondary digital divide, which is mainly characterised by differences in use, is breeding a new round of unequal opportunities with the widespread use of the Internet and new-generation information technologies, and has become an important obstacle to breaking down the urban-rural dichotomy and achieving common prosperity in China.

The widespread use of information technology and the popularity of the Internet as one of the most important sources of contemporary economic growth has become consensus [8,9], but the resulting adverse effects on income distribution have still attracted widespread academic attention [10]. United States productivity data suggest that wages for workers in low-skilled manufacturing will decline as information technology spreads [11]. The process of convergence between information technology and industrialization will create a digital divide, thus leading to the uneven characteristics of urban and rural income growth [12]. Although the implementation of digital economy infrastructure policies has led to a significant increase in Internet penetration in rural areas of China, the "first mover" advantage of groups with earlier access to the Internet may have further widened the Internet dividend gap after the spread of the Internet. The lack of digital literacy and Internet skills in rural areas may also make it difficult for rural residents to enjoy the dividends of the digital economy as much as urban residents [13]. Many scholars have empirically tested the adverse effects of digital economy development on the urban-rural income gap in China, pointing out that the rapid development of the digital economy may cause a new round of unequal in urban-rural wealth distribution [14–16].

In 2013, *the Notice of The State Council on the Issuance of "Broadband China" Strategy and Implementation Plan* issued by The State Council of China clearly pointed out that broadband network is a strategic public infrastructure for economic development in the new era, and it is necessary to further improve its application level, innovate application mode, and expand the new generation of information technology industry. Since the implementation of the "Broadband China" strategy, China has deployed eight years of broadband development goals and paths, laying a solid foundation for comprehensively improving the quality of broadband development, strengthening the digital economy, expanding development space, building a strong network, and sharing digital opportunities. Broadband network has become an indispensable infrastructure to drive industrial upgrading and digital economy development. Based on this, this paper will also take the "Broadband China" strategy as a quasi-natural experiment and evaluate its impact on the urban-rural income gap in China using a multi-period differential model.

Studies have explored the effect of the digital economy on urban-rural income distribution from different perspectives. Still, few studies have explored the mechanism of action in depth. Most studies are based on provincial-level data, making it difficult to capture individual information at the city level. This paper uses panel data of 202 cities from 2011 to 2019 in China to investigate the effect of the digital economy on the urban-rural income gap, the mechanism of impact and spatial spillover characteristics, and discusses possible endogeneity problem using instrumental variables and exogenous shocks quasi-natural experiments.

## 2. Theoretical analysis and research hypotheses

### 2.1 Theories of urban-rural dual structure

According to structuralist development economics, developing nations exhibit a clear urban-rural dual structure phenomena throughout their early stages of development. On the one hand, the vast rural area is still a traditional society before the industrial revolution, and the agricultural sector relies mainly on land and human resources for production. The few cities, on the other hand, are modern society that had a slow industrialization after the advent of colonialism. The industrial sector produces mostly with the use of machines and capital. In developing countries, economic development is largely a result of the reallocation of labour from low-productivity rural to high-productivity urban sectors. The phenomena of urban-rural dual structure will essentially vanish if the labour productivity of urban and rural areas is roughly equal.

The phenomenon of urban-rural dual structure also existed in the early stages of China’s modernization process. The issue is that, after the founding of New China, China concentrated resources on promoting industrialization in an effort to build a modern industrial system quickly, neglecting to promote the conversion of the dual urban-rural structure. In order to obey and serve this strategic intention, the dual system of urban and rural division is gradually established. China’s urban-rural dual system not only prevents the urban-rural dual structure from gradually dissipating with the development of national industrialization, but also makes it worsen and solidify. On the one hand, the urban-rural dual economic structure hasn’t changed consistently with economic growth, exhibiting a pattern of ongoing fluctuation or even phased strengthening [17]. On the other hand, the urban-rural dual economic structure presents spatial imbalance [18]. This motivates academics to investigate the causes and consequences of China’s dual economic structure change. Existing studies are mainly based on the theory of development economics to analyze the dual economic structure of China [19–22]. It not only highlights the government’s industrial bias policy as a significant factor in the widening urban-rural economic divide in China [19], but also contends that the dual economic structure will have a detrimental impact on the country’s economic and social development by affecting resident income distribution, domestic consumption demand, industrial structure upgrading, and social order stability [18]. Most of the above studies take the "Lewis-Fickingham-Lanis model" as the starting point of analysis and regard the labor flow from the traditional sector (represented by rural or agriculture) to the modern sector (represented by urban or non-agricultural) as the main transformation of the dual economic structure. It is pointed out that the dual economic structure is mainly manifested by the income gap between urban and rural residents [23]. At the same time, a number of governance and urban development challenges have emerged as significant barriers preventing China from further improving its social structure and achieving sustainability, and scholars have conducted a great deal of helpful study in this field [24–26].

China’s urban-rural dual structure is also reflected in the uneven distribution of digital resources between urban and rural areas. With the promotion of the strategy of rural revitalization and the construction of digital countryside, digital elements have become an important bridge between urban and rural [27]. At the same time, the digital divide between urban and rural areas still exists, resulting in two groups: the information poor and the information rich. On the one hand, the dual structure leads to significant differences in ICT access between urban and rural residents. Urban residents are faced with "information explosion", even the phenomenon of information backlog or waste; However, the obstacles of the rural residents to absorb the existing information achievements are increasing, the utilization is less, the ability is weak, and even in the state of "information hunger". On the other hand, the economic opportunities of rural residents in a position of information weakness are constantly weakening, as are their wealth creation ability and income level [28], while urban residents in a position of information superiority can more conveniently obtain and utilize digital information through information channels such as the Internet and constantly improve their digital awareness and ability. Eventually, it will lead to the widening of the wealth gap between urban and rural residents and exacerbate the unbalanced development between urban and rural areas.

### 2.2 Effects of digital economy development on urban-rural income gap

Under the background of social digitization, networking and intelligent development, the relationship between China’s digital economy and residents’ income is increasingly close [29]. While enjoying the development dividend brought by the digital economy, the "digital divide" problem caused by it is also worrying. From the perspective of the primary digital divide, although China’s rural Internet penetration rate has increased year by year in recent years, there is still a significant difference between urban and rural areas, and the overall gap between urban and rural Internet penetration rates from 2013 to 2018 is still growing [30]. In terms of the secondary digital divide, due to the lack of application scenarios and usage atmosphere related to the digital economy in rural areas, Internet literacy and usage skills are still at a low level, coupled with the lack of relevant skills training and other factors making the credit system, market system, innovation system and other soft foundations needed for the digital economy still very imperfect. As the relatively disadvantaged side of the economic status, rural Internet users are more likely to use the Internet for entertainment rather than for business transactions and financial gain [31]. In addition, by virtue of their "first mover advantage", urban dwellers could reap the dividends of the digital economy in the early years. Even if the urban-rural divide is gradually bridged, the dividend difference will still widen, and the Matthew effect of "stronger is stronger, weaker is weaker" is difficult to intervene by conventional public policies. Accordingly, research hypothesis 1 of this paper is as follows:

Hypothesis 1: The digital economy will widen the income gap between urban and rural areas.

### 2.3 Impact mechanism of the digital economy development on the urban-rural income gap

Firstly, the Petty-clark theorem summarises the general rule of the industrial structure evolution in which the labour force is transferred from the primary industry to the secondary industry and then to the tertiary industry as the level of economic development and national income increases. The rise of the digital economy has vastly accelerated this process. On the one hand, the digital industry, represented by information transmission, software and information technology services, has exploded in recent years and become a new driving force for economic growth. The value added of information transmission, software and information technology services grew at a compound annual growth rate of 16.22% from 2011 to 2021, significantly higher than the GDP growth rate in the same period. On the other hand, the average salary of information transmission, software and information technology services also leads other industries by a large margin, reaching as high as 25,600 US dollars in 2020, 2.14 times and 3.64 times that of manufacturing and lodging-catering industries, respectively. At the same time, information transmission, software and information technology services are typical technology-intensive and knowledge-intensive industries, which have high requirements for job seekers’ educational background and skill level. The relatively backward education level in rural areas greatly limits the flow of rural labor to high-paying industries with high technical barriers, such as information transmission, software and information technology services, which leads to the widening of the urban-rural income gap.

Secondly, the development of the digital economy is bound to be accompanied by the deepening of the digital finance use, and the difference in the ability of urban and rural residents to obtain economic benefits will impact the urban-rural income gap. The application and popularization of digital finance have greatly facilitated the daily consumption and credit demand of urban and rural residents by creating new financial scenes, increasing people’s availability of financial resources, and playing a significant role in promoting the improvement of the absolute income level of urban and rural residents [32–34]. At the same time, the secondary digital divide is mainly characterised by usage differences, such as the lack of a digital finance usage atmosphere in rural areas, weak learning ability due to farmers’ lower education level, low financial literacy and poor access to financial resources, exists objectively between urban and rural areas. The inertia of payment habits and credit concepts also prevents rural residents from fully enjoying the fruits of digital financial development. It even creates an "information cocoon" and "echo chamber" effect [35]. Although digital finance offers rural residents the possibility to enjoy the same quality of financial services as urban residents, the actual depth of digital finance use by rural residents is significantly lower than that of non-agricultural residents. The penetration rate of digital financial services with large dividend spillover effects such as credit, investment and credit, but with a high threshold of use, is still very low in rural areas. The acceleration of digitalization has led to a widening urban-rural income gap [36], Chinese survey data from 19 poverty-stricken counties and administrative villages show that the proportion of surveyed farmers using third-party payments was only 8.5%, and the purpose of use was mainly online shopping, while more complex service functions such as investment, insurance and online credit were almost unused [14,37]. Accordingly, research hypothesis 2 of this paper is proposed:

Hypothesis 2: The development of the digital economy will push up the share of employment in information services and deepen the use of digital finance, both of which will raise the absolute income level of urban and rural residents, but its pulling effect on the income of urban residents is greater, thus leading to a widening of the urban-rural income gap.

### 2.4 Spatial spillover effects of the digital economy

In contrast to traditional economic behaviour, the digital economy has greatly relaxed the restrictions on time and space for economic activities enabling factors of production such as data and information to be transferred across space at lower costs, leading to a significant increase in the regional spatial correlation and spatial spillover characteristics of economic activities. Many scholars have examined the spatial spillover characteristics of the digital economy from multiple perspectives, such as digital finance and high-quality economic development [38], digital economy and rural revitalization [39], digital economy and urban-rural labor reallocation [40]. Accordingly, the research hypothesis 3 of this paper is proposed:

Hypothesis 3: The impact of the digital economy on the urban-rural income gap has spatial spillover effects.

## 3. Materials and methods

### 3.1 Overview of the study area

**Fig 1:**
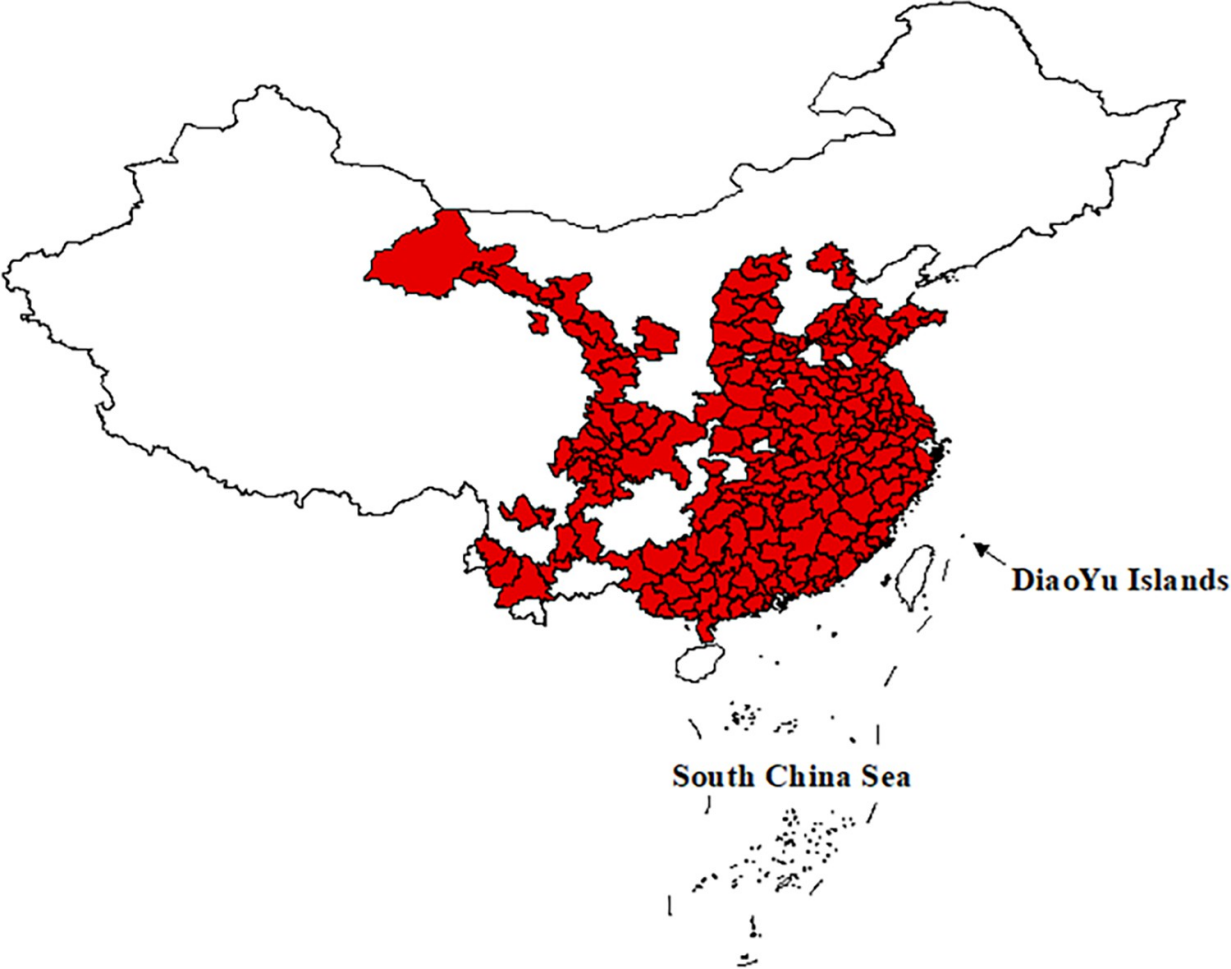
The 202 cities in China studied in this paper.

Based on the availability of data, this paper uses panel data of 202 cities from 2011 to 2019 in China to study the impact of digital economy development on the urban-rural income gap. The distribution of 202 cities is shown in Fig 1.

### 3.2 Variable descriptions

#### 3.2.1 Explained variable: Urban-Rural income gap (*Gap*)

Regarding the selection of indicators for the urban-rural income gap, most studies have used the Thiel index or the urban-rural income ratio to measure it. Among them, the Thiel index takes into account the urban-rural distribution of income as well as the distribution of urban-rural population structure, and is more sensitive to changes in income at the two extremes, while the urban-rural income ratio is more intuitive in an economic sense [41,42]. Based on robustness considerations, the empirical results of both the Thiel Index and the rural-urban income ratio as explained variables will be presented in the main research process of this paper. The calculation formulas of the two indicators are as follows:

$$Gap\_theil_{it}=\sum_{j=1}^{2}\left(P_{ij,t}/P_{i,t}\right)\ln[\left(P_{ij,t}/P_{i,t}\right)/\left(Z_{ij,t}/Z_{i,t}\right)]$$ (1)

$$Gap\_ratio_{it}=P_{iu,t}/P_{ir,t}$$ (2)

Eq (1) is the formula for the Thiel index, where *Gap_theil* is the urban-rural income gap, *P*_*i*,*t*_ is the total income of city *i* in period *t*, *P*_*ij*,*t*_ denotes the income of city *i* urban residents or rural residents in period *t*, *Z*_*i*,*t*_ is the total population of city *i* in period *t*, and *Z*_*ij*,*t*_ denotes the number of people in city *i* urban or rural in period *t*. The smaller the Thiel index, the smaller the difference between urban and rural incomes, and vice versa. Eq (2) is the formula for calculating the urban-rural income ratio, where *Gap_ratio* is the urban-rural income gap, and *P*_*iu*,*t*_ and *P*_*ir*,*t*_ are the incomes of urban residents and rural residents of city *i* in period *t*, respectively.

#### 3.2.2 Core explanatory variable: Digital economy (*Dig*)

Studies on the measurement of the digital economy have focused on different aspects and have not yet formed a relatively uniform standard. Early studies considered the digital economy to be essentially the integration of Internet development with traditional economic activities, and therefore the scale of Internet development was taken as the core indicator for measuring the digital economy [43,44]. Based on reference to existing literature, this paper focuses on the basic components of the digital economy in its measurement. On the basis of Internet development as the core element of the digital economy and the availability of city-level data, the indicators of Internet-related output and smart terminal penetration are incorporated into the measurement system of the digital economy. The relevant variables are: the number of Internet users per 100 people, the total amount of telecommunication services per capita, the total amount of postal services per capita, and the number of mobile phone users per 100 people. The above four variables are dimensionally reduced by means of principal component analysis to finally obtain core explanatory variable *Dig*.

#### 3.2.3 Control variables

In addition to the digital economy, other factors can also impact the urban-rural income gap. In order to minimise the impact of bias on the estimation results due to omitted variables, the following variables are included in the set of control variables by referring to the common practice in the existing literature, mainly: (1) Level of economic development (*Lnpgdp*). The stage of economic development will have an impact on the local wealth distribution and the urban-rural income gap, and this paper uses the logarithm of GDP per capita to indicate the level of economic development. (2) Industrial structure (*Ad*). The evolution of the industrial structure will lead to a redistribution of labour demand, which will impact income distribution. This paper uses the proportion of the tertiary industry in the secondary industry to measure the industrial structure. (3) Fiscal revenue and expenditure ratio (*Pub*). Fiscal revenues and expenditures reflect the fiscal policy preferences of local governments, are also the main means of secondary distribution of national income, and are measured by the ratio of local general budget revenues to local general budget expenditures in this paper. (4) Education expenditure (*Edu*). Education poverty is the "internal cause" of economic and material poverty. This paper uses the proportion of education expenditure in regional GDP to express education expenditure. (5) Financial development level (*Fin*). Expressed as the proportion of deposit and loan balance of financial institutions in local GDP at the end of the year. (6) Social Security Participation (*Sec*). It is expressed by the ratio of the sum of the insured number of urban workers’ basic endowment insurance, the insured number of urban basic medical insurance, the insured number of unemployment insurance to the average local population at the end of the year.

#### 3.2.4 Mediating variable

Information services employment and digital finance use are extremely closely linked to the development of the digital economy. Specifically, the following variables were chosen as path variables for the digital economy to influence the urban-rural income gap. (1) Share of employment in information services (*Infind*). The share of employment in information transmission, software and information technology services, which is most closely related to the development of the digital economy, in relation to the average local population is used. (2) Depth of digital finance use (*Findep*). The depth of use dimension in the Digital Inclusive Finance Index released by Peking University is used to measure the index, which is compiled with the support of massive micro data from the Ant Group Research Institute and can reflect the depth and breadth of digital finance use in various aspects such as daily payments, investment, and credit lending [45]. The depth of use dimension mainly examines the actual use of digital finance services such as payments, money funds, credit, insurance, investment, and it gives greater weight to services with lower penetration and higher thresholds (complexity and riskiness) in the assignment rules.

### 3.3 Data source

Urban-rural income Gap (*Gap*), digital economy (*Dig*), education expenditure (*Edu*) and social security participation (*Sec*) were obtained from the statistical yearbooks of each city. Economic development level (Ln*pgdp*), industrial structure (*Ad*), fiscal revenue and expenditure ratio (*Pub*) and financial development level (*Fin*) were obtained from China City Statistical Yearbook. The depth of digital finance use were obtained from the Peking University Digital Inclusive Finance Index published by the Digital Finance Research Centre of Peking University [45]. The descriptive statistics of the relevant variables are shown in Table 1.

**Table 1:** Descriptive statistics.

	Variable	Obs.	Mean	Std. Dev	Min	Max
**Explained variable**	*Gap_theil*	1,818	0.081	0.045	0.005	0.279
	*Gap_ratio*	1,818	2.384	0.484	1.495	4.626
**Explanatory variable**	*Dig*	1,818	0.000	1.244	-1.741	12.827
**Control variable**	Ln*pgdp*	1,818	10.685	0.593	8.773	12.579
	*Ad*	1,818	0.936	0.474	0.204	5.168
	*Pub*	1,818	0.483	0.226	0.085	1.541
	*Edu*	1,818	0.035	0.018	0.008	0.148
	*Fin*	1,818	2.359	1.097	0.764	11.173
	*Sec*	1,818	0.624	0.779	0.069	8.676
**mediated variable**	*Infind*	1,818	0.002	0.005	0.000	0.062
	*Findep*	1,818	168.389	68.742	12.49	331.958

### 3.4 Research methods

#### 3.4.1 Kernel density estimation

In order to depict the temporal and spatial evolution characteristics of digital economy and urban-rural income gap, this paper will use stata15 software to calculate its kernel density distribution from the perspectives of year and areas.

#### 3.4.2 Benchmark regression

To test the research hypothesis presented above, the empirical model was set up in the following form:

$$Gap\_theil_{it}=\alpha_{0}+\alpha_{1}Dig_{it}+\alpha_{2}Z_{it}+\mu_{i}+\delta_{t}+\varepsilon_{it}$$ (3)

$$Gap\_ratio_{it}=\alpha_{0}+\alpha_{1}Dig_{it}+\alpha_{2}Z_{it}+\mu_{i}+\delta_{t}+\varepsilon_{it}$$ (4)

The above equation is the baseline regression model for this paper, where *i* and *t* denote city and time respectively; *Gap_theil* and *Gap_ratio* are the explained variable urban-rural income gap, calculated using two methods: the Thiel index of urban-rural income and the urban-rural income ratio, respectively; *Dig* is the core explanatory variable digital economy; *Z* is a set of control variables; *μ* is an individual city effect that does not vary over time, *δ* is a time fixed effect, and *ε* denotes an unobservable random disturbance term.

#### 3.4.3 Mechanism test

In addition, to test the possible mechanisms of the direct effects embodied in Eqs (3) and (4), the following model was further set up by introducing the share of employment in information services and the depth of digital finance use into the model [46]:

$$Infind_{it}=\alpha_{0}+\alpha_{1}Dig_{it}+\alpha_{2}Z_{it}+\mu_{i}+\delta_{t}+\varepsilon_{it}$$ (5)

$$Gap_{it}=\alpha_{0}+\alpha_{1}Dig_{it}+\beta Infind_{it}+\alpha_{2}Z_{it}+\mu_{i}+\delta_{t}+\varepsilon_{it}$$ (6)

$$Findep_{it}=\alpha_{0}+\alpha_{1}\ln Dig_{it}+\alpha_{2}Z_{it}+\mu_{i}+\delta_{t}+\varepsilon_{it}$$ (7)

$$Gap_{it}=\alpha_{0}+\alpha_{1}Dig_{it}+\beta Findep_{it}+\alpha_{2}Z_{it}+\mu_{i}+\delta_{t}+\varepsilon_{it}$$ (8)

Where *Indfin* and *Findep* are the mediating variables, indicating the share of employment in information services and the depth of digital finance use, respectively. In the above equations, if the regression coefficients of *Dig* in Eqs (5) and (7) are significant, and the regression coefficients of *Dig* in Eqs (6) and (8) become smaller or no longer significant compared to the baseline model, then *Indfin* and *Findep* are the mediating variables that have an impact on the urban-rural income gap.

#### 3.4.4 Direct examination of absolute income levels

The analysis in the theoretical section mentions that both the digital economy itself and the increase in the share of employment in the information services sector, as well as the deepening use of digital finance, will have a positive effect on the increase in the absolute income levels of urban and rural residents. Then the reason for the widening of the income gap between urban and rural should lie in the different promotion effects of the above three on the income level of urban and rural residents, i.e. the contribution to the absolute income level of urban residents is greater than that of rural residents. In this paper, the following econometric model will be established to examine them:

$$Urban\_inc_{it}=\alpha_{0}+\alpha_{1}X_{it}+\alpha_{2}Z_{it}+\mu_{i}+\delta_{t}+\varepsilon_{it}$$ (9)

$$Rural\_inc_{it}=\alpha_{0}+\alpha_{1}X_{it}+\alpha_{2}Z_{it}+\mu_{i}+\delta_{t}+\varepsilon_{it}$$ (10)

In the above equation, the explained variables *Urban_inc* and *Rural_inc* are urban household disposable income and rural household disposable income, respectively. *X* is the core explanatory variable digital economy (*Dig*) and two mediating variables information service employment proportion (*Infind*) and digital finance usage depth (*Findep*). The remaining variables are consistent with the implications in the baseline model.

#### 3.4.5 Spatial econometric model

Considering that the digital economy has greatly relaxed the constraints of geographical distance on traditional economic activities, it may make it possible for the digital economy to impact the urban-rural income gap in the surrounding regions. In this regard, the following panel spatial econometric model is set up to examine the spatial spillover characteristics of the digital economy:

$$Gap_{it}=\alpha_{0}+\rho WGap_{it}+\gamma WDig_{it}+\alpha_{1}Dig_{it}+\theta WZ_{it}+\alpha_{2}Z_{it}+\mu_{i}+\delta_{t}+\varepsilon_{it}$$ (11)

$$Gap_{it}=\alpha_{0}+\alpha_{1}Dig_{it}+\alpha_{2}Z_{it}+\mu_{i}+\delta_{t}+\varepsilon_{it},\varepsilon_{it}=\lambda W\varepsilon_{it}+u_{it}$$ (12)

Eq (11) is called the Spatial Durbin Model (SDM), where *W* is the spatial weight matrix, *ρ*, *γ*, *θ* are the interaction coefficients of the urban-rural income gap, digital economy, other control variables and the spatial weight matrix *W*, respectively. Eq (12) is the Spatial Error Model (SEM), and *λ* is the coefficient of the interaction term between the random disturbance term and the spatial weight matrix *W*, which represents the possible spatial correlation of unobservable random shocks.

#### 3.4.6 Instrumental variable method

Serious endogeneity problems may lead to inconsistencies in the OLS estimates, thus reducing the credibility of the estimation results. In terms of the research topic of this paper, on the one hand, the formation mechanism of the urban-rural income gap is very complex, and although this paper has controlled for factors that may have an impact on the urban-rural income gap in several aspects, it is still difficult to completely eliminate the impact of omitted variables on the estimation results; On the other hand, the urban-rural income gap will affect the economic aggregate and structure, while regions with higher economic development quality also have a faster digital economy development, which makes the model may have the endogeneity problem of bidirectional causality.

The widespread use of the internet and the development of the digital economy is largely a continuation of dial-up access to fixed telephone lines, and regions with higher fixed-line penetration also tend to have faster digital economic growth. The technology and habits developed in the use of postal services, an important means of delivering information in the pre-Internet era, also influenced the spread and adoption of the digital economy. From this perspective, there is a correlation between the distribution of landline and postal services and the development of the digital economy in the Internet era. In addition, with the rapid development of the Internet, landline and traditional post services have gradually receded from the historical stage, making it difficult to influence the urban-rural income gap in the study period of this paper, thus the exclusivity of the instrumental variables is better met. For these reasons, this paper draws on the method of Huang Qunhui et al and selects historical data on the number of telephone sets and the number of post offices at the end of 1984 as the instrumental variables of the digital economy [47]. As the historical data selected in this paper are cross-sectional data for one year in 1984, they cannot be directly used in the panel analysis. Therefore, by referring to the method of Nunn and Qian, the interaction term between the number of telephone and post office in 1984 and the number of Internet users per 100 people in the previous year is constructed as the instrumental variable of digital economy [48].

#### 3.4.7 Multi-time point DID model

Network infrastructure, as the "information superhighway", is an important support and prerequisite for the development of the digital economy, and the development of the digital economy cannot be achieved without the popularization and improvement of network infrastructure. In order to speed up the informatization process in China, The State Council issued the "Broadband China Strategy and Implementation Plan" in August 2013, which put forward the phased development goals of basically realizing optical fiber to buildings and households in urban and broadband to villages in rural in 2015.The Ministry of Industry and Information Technology (MIIT) and the National Development and Reform Commission (NDRC) identified 117 cities (city clusters) as "broadband China" demonstration cities (city clusters) in three batches in 2014, 2015 and 2016. In order to further test the robustness of the research results of this paper and eliminate the interference of the endogenous factors not considered in the research results, this paper introduced the exogenous shock of the "broadband China" strategy, and used the multi-time point DID model to evaluate its impact on the urban-rural income gap realistically, and set the research model as follows:

$$Gap_{it}=\alpha_{0}+\alpha_{1}Event_{it}+\alpha_{2}Z_{it}+\mu_{i}+\delta_{t}+\varepsilon_{it}$$ (13)

$$Med_{it}=\alpha_{0}+\alpha_{1}Event_{it}+\alpha_{2}Z_{it}+\mu_{i}+\delta_{t}+\varepsilon_{it}$$ (14)

$$Gap_{it}=\alpha_{0}+\alpha_{1}Event_{it}+\beta Med_{it}+\alpha_{2}Z_{it}+\mu_{i}+\delta_{t}+\varepsilon_{it}$$ (15)

In the above equation, *Event* is a dummy variable for exogenous event shocks, indicating whether an individual city was included in the “Broadband China” pilot in that year, taking 1 if it was included and 0 if it was not; *Med* is the share of employment in information services (*Infind*) and depth of digital finance use (*Findep*), the two mediating variables mentioned earlier; *Z* is a set of control variables with the same meaning as above. In the absence of the exogenous event of “Broadband China”, the treatment and control samples should have approximately the same trend. For this reason, this paper refers to the processing method provided by Jacobson et al [49], and sets the following parallel trend test model:

$$Gap_{it}=\alpha_{0}+\alpha_{1}\sum_{t=-2}^{5}Period_{i,s}+\alpha_{2}Z_{it}+\mu_{i}+\delta_{t}+\varepsilon_{it}$$ (16)

Where *Period* is a dummy variable, which represents the *s* year when the sample cities are included in the "Broadband China" strategy. *s* = 0 represents the year of policy implementation, namely the base period; A negative value of *s* indicates the *s* years before the implementation of the "broadband China" strategy; If *s* is positive, it means *s* years after the implementation of the "Broadband China" strategy. If the null hypothesis that the coefficient of *Period* is equal to 0 cannot be rejected at the significance level when *s*<0 and the coefficient of *Period* is gradually different from 0 after *s*>0, it means that the parallel trend test has been passed.

## 4. Empirical results

### 4.1 Spatio-temporal characteristic analysis

#### 4.1.1 Spatio-temporal characteristic analysis of digital economy

China’s digital economy has rapidly developed thanks to the widespread popularization of the Internet and intelligent terminals, as well as the launch of a series of policies such as "Broadband China." From 2017 to 2021, the scale of China’s digital economy increased from US $3.78 trillion to US $6.32 trillion, ranking the second in the world and becoming one of the main engines driving economic growth. At the same time, due to the historical origin, resource endowment, and the unbalanced development strategy of economic policy center offset to the east after the reform and opening up, China has the status quo of regional imbalance for a long time.

**Fig 2:**
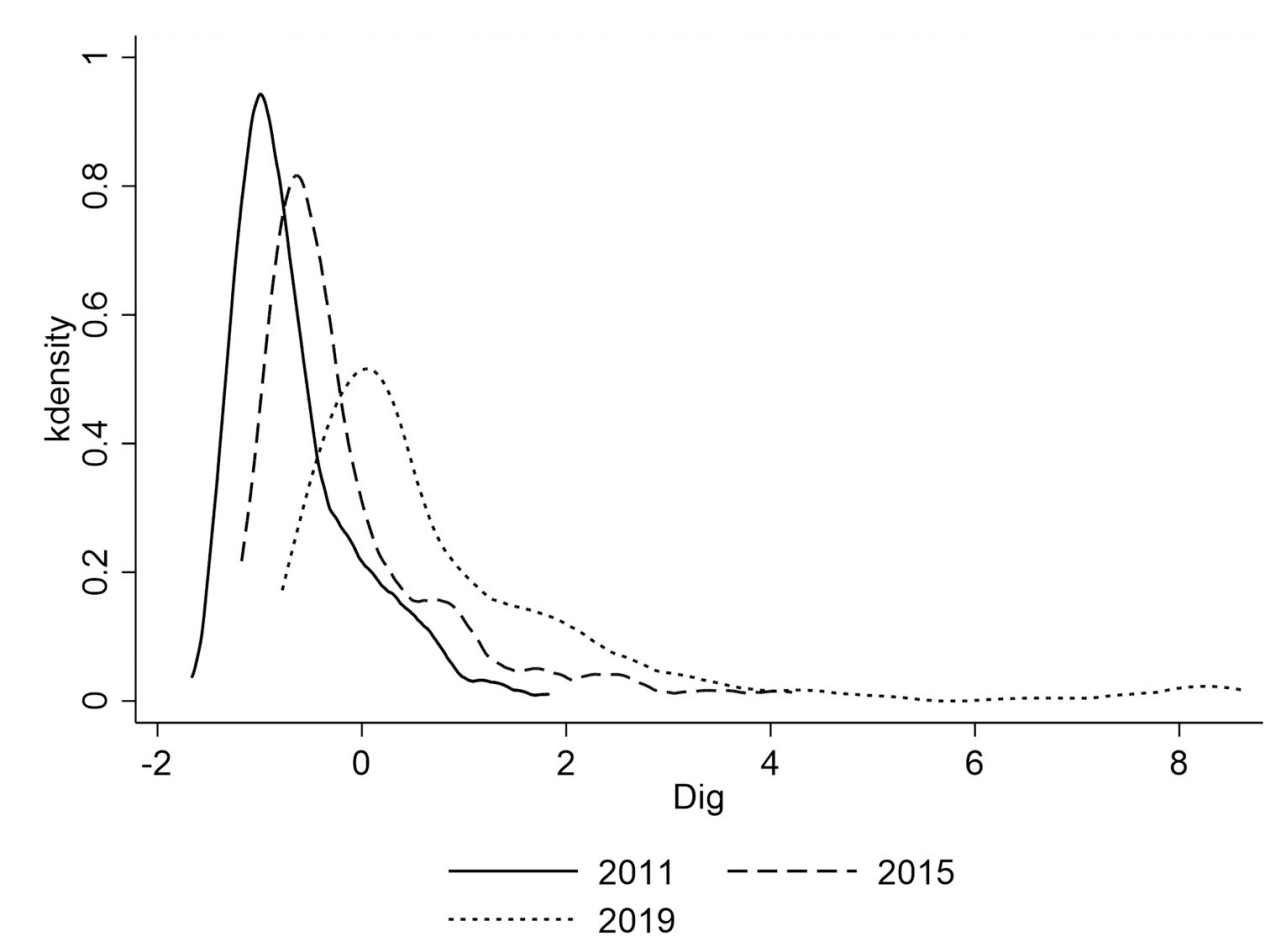
Kernel density estimation of the digital economy by year.

**Fig 3:**
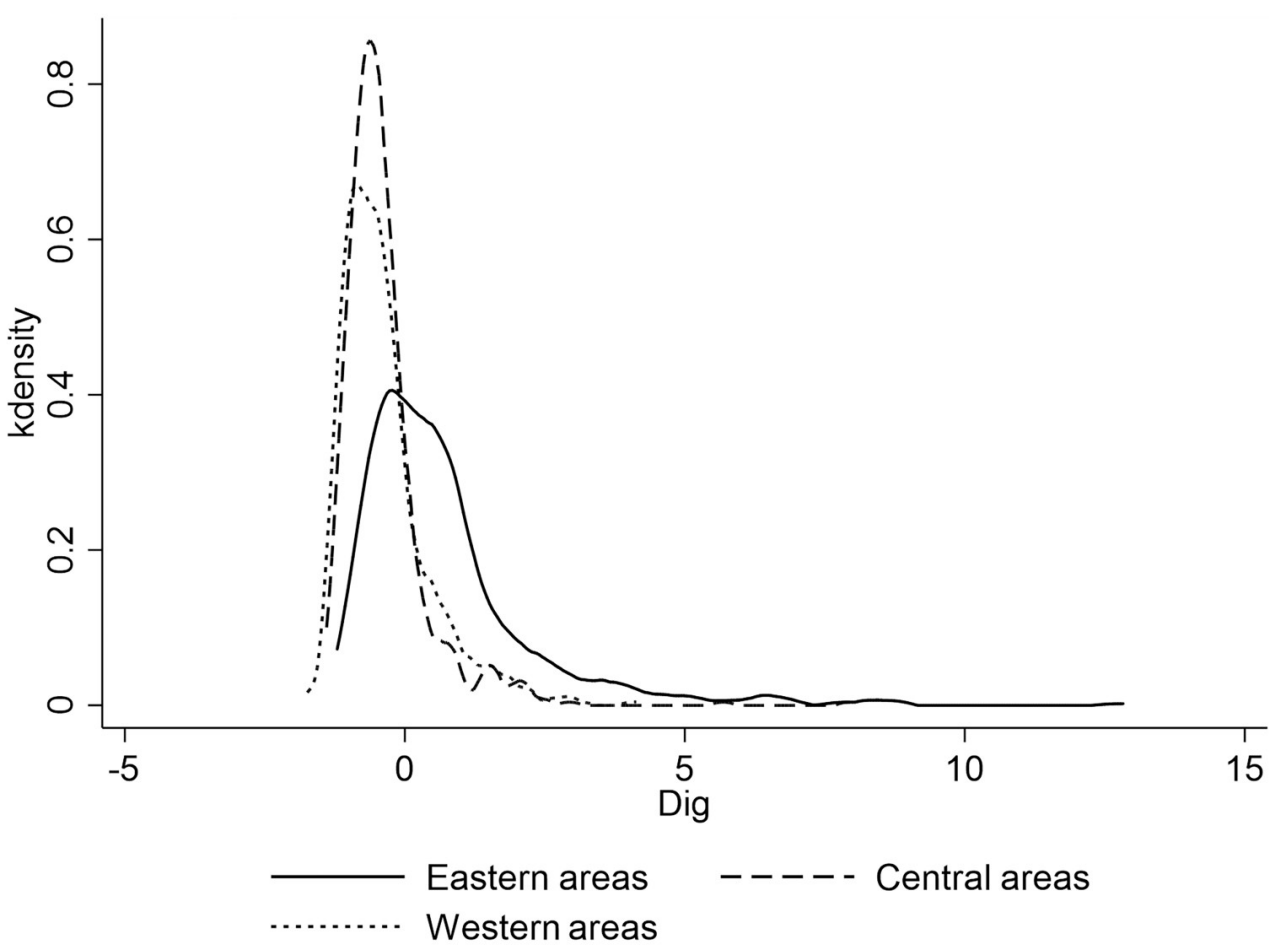
Kernel density estimation of the digital economy by areas.

Figs 2 and 3 show annual kernel density estimates and areas kernel density estimates of digital economy development in China, respectively. As can be seen from Fig 2, the digital economy index corresponding to the peak of kernel density estimation in 2019 is larger than that in 2015, which is significantly larger than that in 2011, indicating that the development of China’s digital economy shows a steady rise. Additionally, it is evident that the digital economy index’s level of concentration is declining over time. As can be seen from Fig 3, the digital economy development index in eastern areas is the highest, concentrated around 0 value, while the distribution characteristics of digital economy development in central and western areas are highly coincidently (Tibet, Xinjiang, Ningxia and Qinghai are not included in the study’s scope due to a severe lack of data, and the digital economy tends to be developing slowly in these areas. The fact that the distribution characteristics of the central and western areas are so similar may be due to this), and the development level of digital economy in both two areas is significantly lower than that in the eastern areas.

#### 4.1.2 Spatio-temporal characteristic analysis of Urban-rural income gap

**Fig 4:**
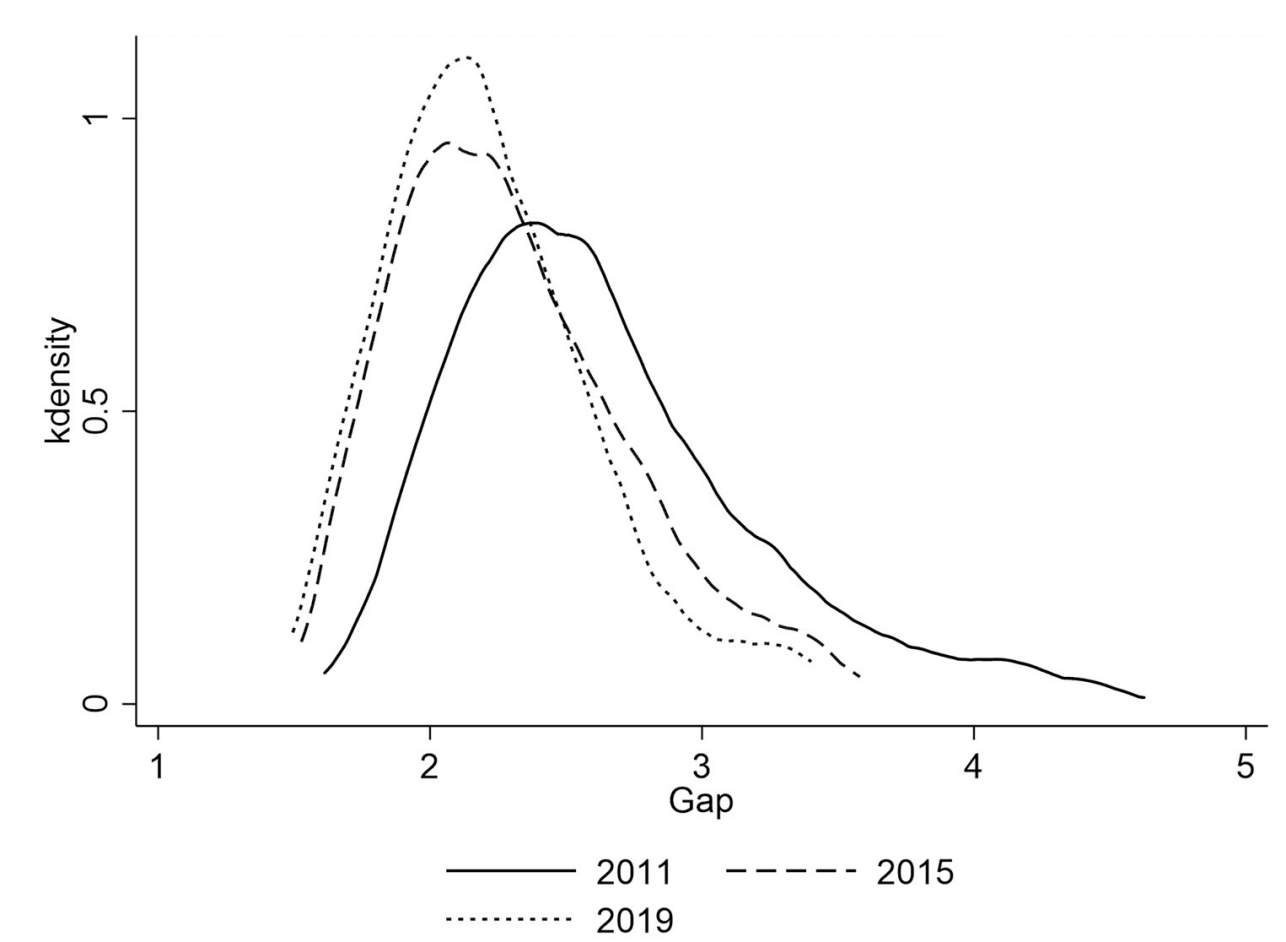
Kernel density estimation of the urban-rural income gap by year.

**Fig 5:**
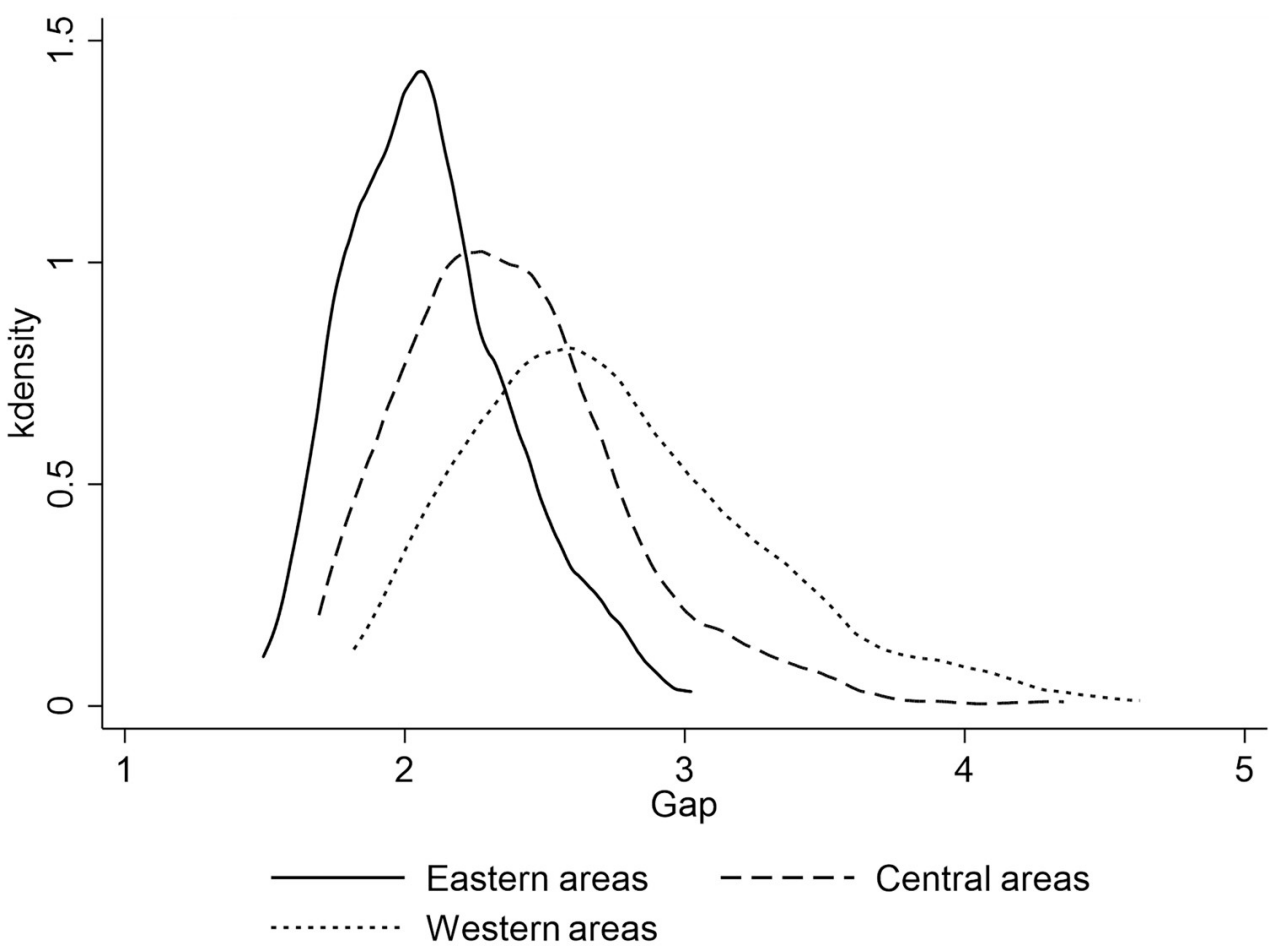
Kernel density estimation of the urban-rural income gap by areas.

Fig 4 shows kernel density estimates of China’s urban-rural income gap (measured by urban-rural income ratio) in 2011, 2015, and 2019. It can be seen that the urban-rural income gap corresponding to the wave peak in 2011 is significantly larger than that in 2015 and 2019, and the distribution in 2015 and 2019 is highly similar. This indicates that the narrowing of urban-rural income gap in China mainly occurred before 2015, and barely changed after 2015. Fig 5 shows three areas kernel density estimation of the urban-rural income gap in China, which shows obvious regularity, the income gap between urban and rural in the three areas of China increases successively along the direction of the eastern areas, the central areas and the western areas, which is also consistent with the regional development level of China. In addition, it can be seen from the shape of the estimation results that the aggregation degree of urban-rural income gap is also decreases along the direction of eastern areas, central areas and western areas, that is, the urban-rural income gap is more concentrated in the eastern areas, followed by the central areas and the western areas.

### 4.2 Benchmark regression

Table 2 reports the estimation results of the benchmark regression. Columns (1) and (2) do not add control variables, columns (3) and (4) further introduce the level of economic development into the regression, and columns (5) and (6) show the results after adding all control variables. As can be seen, except for the regression coefficient of *Dig* in column (2), which is not significant, the coefficients of *Dig* in all other columns are positive at a significance level below 5%, whether *Gap_theil* is used as the explanatory variable or *Gap_ratio* is used as the explanatory variable, indicating that the development of the digital economy has widened the urban-rural income gap and exacerbated the urban-rural income " Matthew effect". Among the control variables, the regression coefficient of the level of economic development (*Lnpgdp*) is significantly negative at the 1% level of significance, indicating that the increase in the level of economic development is conducive to the reduction of the urban-rural income gap; the coefficient of the education expenditure (*Edu*) is also positive at the 1% level of significance, presumably because the investment in education has increased in favour of rural areas in recent years, thus contributing to the reduction of the urban-rural income gap. The remaining control variables did not have a significant effect on the urban-rural income gap. Thus, the research hypothesis hypothesis 1 of this paper is confirmed.

**Table 2:** Benchmark regression results.

Variable	*Gap_theil*	*Gap_ratio*	*Gap_theil*	*Gap_ratio*	*Gap_theil*	*Gap_ratio*
	(1)	(2)	(3)	(4)	(5)	(6)
*Dig*	0.00172**	0.00825	0.00258***	0.0196**	0.00284***	0.0244**
	(0.000748)	(0.00727)	(0.000767)	(0.00900)	(0.000768)	(0.00939)
Ln*pgdp*			-0.0183***	-0.241***	-0.0223***	-0.307***
			(0.00436)	(0.0545)	(0.00481)	(0.0626)
*Ad*					-0.00518	-0.0569
					(0.00374)	(0.0459)
*Pub*					-0.0217**	-0.0210
					(0.00862)	(0.0811)
*Edu*					-0.318***	-3.594***
					(0.108)	(1.324)
*Fin*					-0.00000063	-0.00589
					(0.000903)	(0.00854)
*Sec*					-0.00120	0.0617*
					(0.00343)	(0.0343)
*Constant*	0.106***	2.641***	0.296***	5.141***	0.363***	5.975***
	(0.00159)	(0.0154)	(0.0454)	(0.568)	(0.0512)	(0.682)
Year FE	Yes	Yes	Yes	Yes	Yes	Yes
City FE	Yes	Yes	Yes	Yes	Yes	Yes
Number of city	202	202	202	202	202	202
Observations	1,818	1,818	1,818	1,818	1,818	1,818
within-R^2^	0.585	0.580	0.594	0.595	0.604	0.605

Note

***, **, * indicate significance at the levels of 1%, 5%, and 10%, respectively. The same applies to the following table.

### 4.3 Mechanism test

The above analysis theoretically illustrates the two transmission mechanisms of the digital economy’s impact on the urban-rural income gap. Here, hypothesis 2 of this paper is verified. Table 3 shows the estimation results with the share of employment in information services (*Infind*) as the mediating variable. As can be seen from column (1), the coefficient of the digital economy on the mediating variable is significantly positive at the 1% level of significance, indicating that the digital economy increases the share of employment in information services. In the regression results with *Gap_theil* as the explained variable, after introducing *Infind* into the model for estimation again, the coefficient of *Dig* for the digital economy in column (3) is 0.00199, which is smaller than the coefficient of *Dig* in column (2), and the regression coefficient of the mediating variable *Infind* is still significant. The results of the regression with *Gap_ratio* as the explained variable are similar. After introducing *Infind* into the model again, the coefficient of *Dig* in column (5) is significantly smaller than the coefficient of *Dig* in column (4) and is no longer significant, suggesting that the increase in the share of employment in information services is one of the mechanisms through which the development of the digital economy widens the income gap between urban and rural areas.

**Table 3:** Mechanism test: Mediating variable is *Infind*.

Variable	*Infind*	*Gap_theil*	*Gap_theil*	*Gap_ratio*	*Gap_ratio*
	(1)	(2)	(3)	(4)	(5)
*Dig*	0.000883***	0.00284***	0.00199***	0.0244**	0.0135
	(0.000201)	(0.000768)	(0.000741)	(0.00939)	(0.00966)
*Infind*			0.967**		12.34**
			(0.457)		(4.902)
*Constant*	0.00918	0.363***	0.354***	5.975***	5.862***
	(0.00919)	(0.0512)	(0.0513)	(0.682)	(0.694)
*Controls*	Yes	Yes	Yes	Yes	Yes
Year FE	Yes	Yes	Yes	Yes	Yes
City FE	Yes	Yes	Yes	Yes	Yes
Number of city	202	202	202	202	202
Observations	1,818	1,818	1,818	1,818	1,818
within-R^2^	0.204	0.604	0.609	0.609	0.612

Table 4 presents the estimation results with depth of digital finance use as a mediating variable. Similar to the analysis above, the regression coefficient of the digital economy on the mediating variable digital finance (*Findep*) in column (1) is positive at the 1% significance level, thus validating the contribution of the digital economy to the depth of digital finance use. By looking at the coefficient values of the core explanatory variable *Dig* and its change in significance, it can be seen that: the regression coefficients of the digital economy on the urban-rural income gap in columns (3) and (5) have decreased or are less significant compared to columns (2) and (4), and the regression coefficient of the mediating variable *Findep* is still significant, indicating that the depth of digital finance use is another pathway for the impact of the digital economy on the urban-rural income gap variable. Thus, the research hypothesis 2 of this paper is confirmed.

**Table 4:** Mechanism test: Mediating variable is *Findep*.

Variable	*Findep*	*Gap_theil*	*Gap_theil*	*Gap_ratio*	*Gap_ratio*
	(1)	(2)	(3)	(4)	(5)
*Dig*	2.306***	0.00284***	0.00251***	0.0244**	0.0197**
	(0.505)	(0.000768)	(0.000757)	(0.00939)	(0.00949)
*Findep*			0.000143**		0.00203***
			(0.0000695)		(0.000659)
*Constant*	80.30***	0.363***	0.351***	5.975***	5.812***
	(27.59)	(0.0512)	(0.0494)	(0.682)	(0.673)
*Controls*	Yes	Yes	Yes	Yes	Yes
Year FE	Yes	Yes	Yes	Yes	Yes
City FE	Yes	Yes	Yes	Yes	Yes
Number of city	202	202	202	202	202
Observations	1,818	1,818	1,818	1,818	1,818
within-R^2^	0.988	0.604	0.607	0.605	0.610

### 4.4 Direct examination of absolute income levels

Table 5 shows that the regression coefficients of *Dig*, *Infind* and *Findep* are all positive at the 1% level of significance, indicating that both the three significantly contribute to the increase in absolute income of urban and rural residents. However, the regression coefficients in *Urban_inc* are significantly larger than those in *Rural_inc*, indicating that these three factors have a greater contribution to the absolute income level of urban residents but a smaller contribution to the absolute income level of rural residents, which provides a more intuitive perspective to explain the widening of the urban-rural income gap by the development of the digital economy.

**Table 5:** A direct examination of absolute income levels of urban and rural residents.

	explanatory variable is *Dig*		explanatory variable is *Infind*		explanatory variable is *Findep*	
Variable	*Urban_inc*	*Rural_inc*	*Urban_inc*	*Rural_inc*	*Urban_inc*	*Rural_inc*
	(1)	(2)	(3)	(4)	(5)	(6)
*Dig*	0.0843***	0.0528***				
	(0.0233)	(0.0125)				
*Infind*			43.03***	19.41***		
			(9.647)	(3.659)		
*Findep*					0.00488***	0.00302***
					(0.00116)	(0.000618)
*Constant*	3.625***	1.673**	2.362**	0.826	1.863**	0.571
	(0.830)	(0.683)	(0.978)	(0.634)	(0.901)	(0.630)
*Controls*	Yes	Yes	Yes	Yes	Yes	Yes
Year FE	Yes	Yes	Yes	Yes	Yes	Yes
City FE	Yes	Yes	Yes	Yes	Yes	Yes
Number of city	202	202	202	202	202	202
Observations	1,818	1,818	1,818	1,818	1,818	1,818
within-R^2^/ centered-R^2^	0.924	0.914	0.929	0.914	0.922	0.911

### 4.5 Analysis of spatial spillover effects

Before conducting the spatial analysis, the spatial autocorrelation effects of the digital economy and the urban-rural income gap were first tested. Table 6 reports the results of the tests of the spatial autocorrelation effects based on the Moran’I index for each year of the study period under the adjacency matrix, the geographic matrix and the economic matrix. From the results, it can be seen that the Moran’I indices for the digital economy and the urban-rural income gap for the 2011–2019 are all significantly positive at the 1% significance level, indicating that digital economy and the urban-rural income gap have significant "positive-positive" clustering characteristics during the study period.

**Table 6:** Moran index test for spatial correlation.

	*Gap_theil*			*Dig*		
	adjacency matrix	geographic matrix	economic matrix	adjacency matrix	geographic matrix	economic matrix
2011	0.511***	0.101***	0.296***	0.412***	0.102***	0.524***
2012	0.489***	0.102***	0.292***	0.420***	0.093***	0.468***
2013	0.559***	0.119***	0.390***	0.453***	0.106***	0.321***
2014	0.578***	0.136***	0.368***	0.396***	0.097***	0.427***
2015	0.541***	0.114***	0.351***	0.412***	0.094***	0.411***
2016	0.549***	0.115***	0.354***	0.409***	0.091***	0.403***
2017	0.551***	0.117***	0.356***	0.323***	0.082***	0.366***
2018	0.548***	0.118***	0.356***	0.253***	0.067***	0.287***
2019	0.550***	0.119***	0.355***	0.194***	0.056***	0.290***

Table 7 shows the results of the LM test for spatial regression model selection, which shows that the LM- lag, Robust LM- lag, LM- error, Robust LM- error are all significant at and below the 5% significance level under the adjacency and geographic matrices, indicating that the test results significantly reject the null hypothesis that there is no spatial lag effects and spatial error effects, and the Spatial Dubin Model should be used for estimation. In the test results based on the economic matrix, both LM-error and Robust LM-error rejected the null hypothesis of no spatial error effects at the significance level of 5%. However, LM-lag and Robust LM-lag could not reject the null hypothesis that there is no spatial lag effects at the significance level of 10%, so the Spatial Error Model was selected for estimation under the economic matrix. The above results again indicate the necessity of spatial econometric analysis.

**Table 7:** LM test for model selection.

	adjacency matrix		geographic matrix		economic matrix	
	LM statistics	P values	LM statistics	P values	LM statistics	P values
LM-lag	10.209	0.001	6.042	0.014	2.210	0.137
Robust LM- lag	16.138	0.000	23.214	0.000	0.173	0.677
LM-error	159.370	0.000	114.702	0.000	6.313	0.012
Robust LM- error	165.300	0.000	131.874	0.000	4.277	0.039

Table 8 reports the results of the spatial regression of spatio-temporal two-way fixed effects under the three matrices. It can be seen that the development of digital economy not only widens the urban-rural income gap within the region, but also widens the urban-rural income gap in the surrounding areas. In the adjacency matrix and geographic matrix, the spatial interaction term *ρ* of the explained variables is significantly positive at the significance level of 1%, which again verifies the positive spatial autocorrelation characteristic of "high clustering-low clustering" of the urban-rural income gap. The spatial interaction coefficient *λ* of the random error terms under the economic matrix is also positive at the significance level of 1%, indicating that the spatial correlation among regions is also reflected in the systematic error shock. When the coefficient of the spatial lag term of the explained variable urban-rural income gap is significantly not 0, the existence of the "feedback effect" makes it difficult to accurately measure the spatial spillover effect of the urban-rural income gap directly through the coefficient of the Spatial Durbin Model, and the method of partial differential effect decomposition should be used to measure it. As can be seen in Table 8, the direct effect, indirect effect and total effect of digital economy on the urban-rural income gap under adjacency matrix and geographical matrix are all significantly positive, which again confirms the spatial interaction effects between digital economy and urban-rural income gap. Thus, hypothesis 3 of this paper is verified.

**Table 8:** Estimated results of spatial spillover effects.

Variable	*Gap_theil*			*Gap_ratio*		
Matrix types	adjacency matrix	geographic matrix	economic matrix	adjacency matrix	geographic matrix	economic matrix
	(1)	(2)	(3)	(4)	(5)	(6)
*Dig*	0.00111	0.00174**	0.00257***	0.0125	0.0165**	0.0168***
	(0.000677)	(0.000707)	(0.000542)	(0.00777)	(0.00761)	(0.00564)
W× *Dig*	0.00348***	0.0286***		0.0251**	0.287***	
	(0.00103)	(0.00601)		(0.0123)	(0.0667)	
*ρ*	0.490***	0.851***		0.456***	0.880***	
	(0.0457)	(0.0233)		(0.0445)	(0.0197)	
*λ*			0.165***			0.271***
			(0.0429)			(0.0395)
*Controls*	Yes	Yes	Yes	Yes	Yes	Yes
Year FE	Yes	Yes	Yes	Yes	Yes	Yes
City FE	Yes	Yes	Yes	Yes	Yes	Yes
direct effect	0.00173**	0.00277***		0.0169*	0.0295***	
	(0.000714)	(0.000720)		(0.00866)	(0.00802)	
indirect effect	0.00718***	0.202***		0.0522**	2.534***	
	(0.00173)	(0.0454)		(0.0217)	(0.655)	
total effect	0.00891***	0.204***		0.0691**	2.563***	
	(0.00203)	(0.0455)		(0.0272)	(0.658)	
Number of city	202	202	202	202	202	202
Observations	1,818	1,818	1,818	1,818	1,818	1,818
within-R^2^	0.4704	0.0766	0.4563	0.5328	0.4192	0.5138
LogL	5670.7750	5573.3998	5484.8271	1395.7575	1349.5123	1242.9770

## 5. Robustness tests

### 5.1 Replace the explained variable

The existing literature mainly measures the urban-rural income gap by Theil index and urban-rural income ratio. In the main process of empirical analysis, this paper not only reports the estimation results of Theil index (*Gap_theil*) as the explained variable but also reports the estimation results of *Gap_ratio* as the explained variable. As can be seen from the previous analysis, in the main analysis process, the two have a high degree of consistency in the presentation of results, indicating that the research conclusions of this paper are robust.

### 5.2 Alleviate the impact of macro factors

Although the fixed effects of city and time dimensions are controlled in this study, the individual effects that vary from year to year as unobservable heterogeneous shocks may still interfere with the estimation results in this paper. Referring to the practice of Zhao et al [50], the fixed effects of provinces and the interactive fixed effects of provinces and years are added to the model to control the changes of unobserved macroscopic and systematic factors caused by the extensive development of digital economy. Columns (1) and (2) in Table 9 show the estimation results after adding the province fixed effect and its interaction effect with year. It can be seen that the coefficient of digital economy Dig is still significantly positive.

**Table 9:** Robustness tests.

	alleviate the impact of macro factors		Instrumental variable	
Variable	*Gap_theil*	*Gap_ratio*	*Gap_theil*	*Gap_ratio*
	(1)	(2)	(3)	(4)
*Dig*	0.00279***	0.0242**	0.00718**	0.123***
	(0.000762)	(0.00943)	(0.00316)	(0.0397)
*Constant*	-2.060*	-2.936	0.395***	7.765***
	(1.137)	(13.06)	(0.0706)	(0.897)
*Controls*	Yes	Yes	Yes	Yes
Year FE	Yes	Yes	Yes	Yes
City FE	Yes	Yes	Yes	Yes
Province FE	Yes	Yes	No	No
Province × Year	Yes	Yes	No	No
Kleibergen-Paap rk LM Statistics			16.068	16.068
			[0.0003]	[0.0003]
Cragg-Donald Wald F Statistics			26.682	26.682
			{8.68}	{8.68}
Kleibergen-Paap rk Wald F Statistics			9.722	9.722
			{8.68}	{8.68}
Hansen J Statistics			1.323	0.728
			[0.2500]	[0.3937]
Number of city	202	202	161	161
Observations	1,818	1,818	1,449	1,449
within-R^2^/ centered-R^2^	0.609	0.606	0.912	0.912

Note: The value in [] is the P-value of the statistic, and the value in {} is the critical value at the 10% level of the weak identification test.

### 5.3 Instrumental variable method

Columns (3) and (4) of Table 9 report the results of the estimation using the Limited Information Maximum Likelihood (LIML) method, which is more insensitive to weak instrumental variables, and it can be seen that the regression coefficients of the digital economy *Dig* are all positive at the 1% level of significance, in line with the estimation results of the previous benchmark regression. In addition, in a series of tests on instrumental variables, the Kleibergen-Paap RK LM statistic was significant at the significance level of 1%, indicating that the test results strongly rejected the null hypothesis of unidentification. The Cragg-Donald Wald F statistic and Kleibergen-Paap RK Wald F statistic are both greater than the 10% critical value of 8.68, indicating that the model does not have the problem of weak instrumental variables. The P value of Hansen J statistic is greater than 0.1, indicating that the test result cannot reject the null hypothesis that "all instrumental variables are exogenous".

### 5.4 Exogenous shocks based on "Broadband China" strategy

Table 10 shows the estimated results of the regression of Eq (16). It can be seen that the coefficient of the dummy variable *Period* before the implementation of the policy could not reject the null hypothesis of equal to 0 at the 10% significance level, while the regression coefficient of *Period* after the implementation of the policy is gradually significant and the p-value is getting smaller, indicating that the data of this study satisfies the parallel trend test.

**Table 10:** Parallel trend test.

	*Period_-2*	*Period_-1*	*Period_0*	*Period_1*	*Period_2*	*Period_3*	*Period_4*	*Period_5*
*Coefficients*	0.002442	0.004099	0.006064	.0077877	0.0087885	0.0095838	0.0110787	0.0132709
P value	0.378	0.174	0.051	0.014	0.008	0.006	0.004	0.002

Columns (2) and (5) in Table 11 show the results of estimating Eq (13), which shows that the coefficient of the exogenous shock *Event* is positive at the 1% level of significance, indicating that the implementation of the "broadband China" strategy significantly widens the urban-rural income gap. Columns (1) and (4) are the results of estimating Eq (14), and the coefficient of *Event* is positive at the 1% level of significance, indicating that the implementation of the "broadband China" strategy has significantly contributed to both mediating variables. By comparing columns (2) (3) and (5) (6), it can be found that the regression coefficients of exogenous shock *Event* decreased after the model introduced mediation variables, and the regression coefficients of the two mediation variables were both positive at the significance level of 5%. It shows that *Infind* and *Findep* are the two mechanisms of "Broadband China" strategy to widen the urban-rural income gap. This paper also estimates the model with *Gap_ratio* as the explained variable, and the results are consistent with those in Table 11.

**Table 11:** Test results of "Broadband China" exogenous shock.

	Mediation variable is *Infind*			Mediation variable is *Findep*		
变量	*Infind*	*Gap_theil*	*Gap_theil*	*Findep*	*Gap_theil*	*Gap_theil*
	(1)	(2)	(3)	(4)	(5)	(6)
*Event*	0.000985***	0.00638***	0.00536**	3.110***	0.00638***	0.00591***
	(0.000261)	(0.00220)	(0.00226)	(0.960)	(0.00220)	(0.00213)
*Infind*			1.039**			
			(0.451)			
*Findep*						0.000151**
						(0.0000677)
*Constant*	-0.00986	0.293***	0.304***	29.23	0.293***	0.289***
	(0.00730)	(0.0490)	(0.0499)	(23.96)	(0.0490)	(0.0480)
*Controls*	Yes	Yes	Yes	Yes	Yes	Yes
Year FE	Yes	Yes	Yes	Yes	Yes	Yes
City FE	Yes	Yes	Yes	Yes	Yes	Yes
Number of city	202	202	202	202	202	202
Observations	1,818	1,818	1,818	1,818	1,818	1,818
within-R^2^	0.138	0.605	0.611	0.988	0.605	0.608

**Fig 6:**
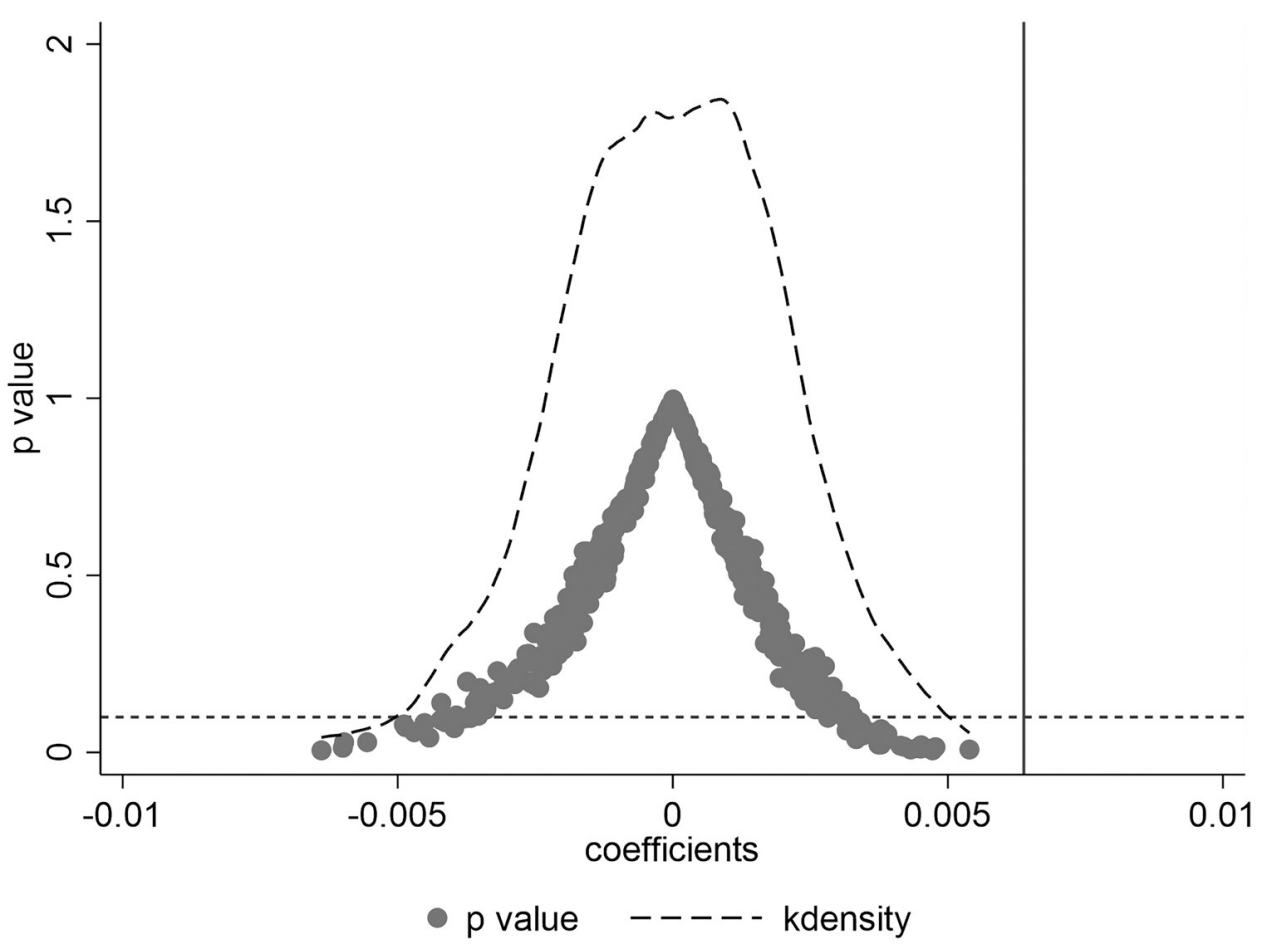
Placebo test.

Although tests based on exogenous event shocks can largely avoid possible endogeneity problems, omitted variables that are not considered may still interfere with the results. According to Cai et al. and Zhong et al. [51,52], cities were randomly selected from the sample as the treatment group entering the "Broadband China" pilot to re-estimate Eq (13). The above process was repeated 500 times, and the estimated coefficient of the placebo test was finally obtained. Fig 6 shows the distribution of regression coefficients after 500 repeated sampling. It can be seen that the regression coefficients are highly similar to the normal distribution of 0 mean, and the benchmark regression coefficient of the model, 0.00638, is significantly anomaly in the coefficient distribution of the placebo test, which indicates to a certain extent that unobservible random factors do not significantly interfere with the study.

## 6. Discussion

### 6.1 Study innovations and significance

The digital divide caused by the development of digital economy has received more and more attention around the world. Based on this background, this paper discusses in-depth the connection between the urban-rural income gap and the digital economy, offering some insights in the following three areas. First, on the basis of examining the direct impact of digital economy on the urban-rural income gap, the possible mechanism of action is also tested. Second, considering that the digital economy eliminates the geographical restrictions of the traditional economy, the spatial spillover characteristics of the digital economy is verified. Third, the quasi-natural experiment of "Broadband China" strategy is introduced, and the multi-time point did method is used to verify the main research conclusions of this paper again.

This study can help academia and government better understand how the digital economy affects the urban-rural income gap by examining its causes, mechanisms, and spatial characteristics.

### 6.2 Countermeasures and suggestions

Based on the main findings of this paper, the following policy implications emerge: firstly, in addition to further improving the hardware facilities of the digital economy in rural areas, government should focus on digital literacy and skills training for rural residents to make the development of the digital economy more inclusive. Secondly, the poor level of education in rural areas makes it difficult for rural residents to find employment in high-paying industries such as information services, which are closely related to the digital economy, due to a lack of relevant skills. Only by increasing investment in education in rural areas and addressing the underlying "internal causes” of educational poverty can the dividends of economic development, including the digital economy, truly reach the majority of farmers. Thirdly, the government should make full use of the spatial spillover characteristics of the digital economy and study the introduction of relevant inclusive policies, so as to increase the radiation of the digital economy from regional central cities to other cities and rural areas.

### 6.3 Outlook

Due to data availability, author ability and other reasons, some topics have not been further expanded, such as explaining the income effect of the digital economy from a micro perspective, and whether the "information cocoon room" plays a role in it. we leaving these challenging but interesting topics for future research.

## 7. Conclusion

With the development of digital economy, the primary digital divide characterized by accessibility is gradually being bridged. At the same time, the secondary digital divide, characterised mainly by usage differences, is breeding a new round of urban-rural inequality as the use of Internet technology spreads. Based on the issue of opportunity inequality and income gap between urban and rural areas that may result from the extensive development of the digital economy, this paper uses panel data from 2011–2019 in China to conduct a study in this regard, and the main conclusions are as follows: (1) A benchmark regression based on a panel fixed effects model shows that the development of the digital economy has significantly exacerbated the ’Matthew effect’ on urban-rural incomes, leading to a further widening of the urban-rural income gap. (2) The rising share of employment in information services and the deepening use of digital finance may be two important mediating mechanisms. (3) On the investigation to the absolute income level also supports this conclusion. digital economy and the two mediating variable have a greater contribution to the absolute income level of urban residents but a smaller contribution to the absolute income level of rural residents. (4) Further analysis shows that the widening effect of digital economy on urban-rural income gap still has significant spatial spillover characteristics. (5) It is found that the main research conclusions of this paper are still robust after testing with the methods of replacing explained variables, instrumental variables and exogenous shock test.

## Supporting information

S1 Data (XLSX)
